# High-Flow Oxygen Therapy in the Perioperative Setting and Procedural Sedation: A Review of Current Evidence

**DOI:** 10.3390/jcm12206685

**Published:** 2023-10-23

**Authors:** Lou’i Al-Husinat, Basil Jouryyeh, Ahlam Rawashdeh, Abdelrahman Alenaizat, Mohammad Abushehab, Mohammad Wasfi Amir, Zaid Al Modanat, Denise Battaglini, Gilda Cinnella

**Affiliations:** 1 Department of Clinical Medical Sciences, Faculty of Medicine, Yarmouk University, Irbid 21163, Jordan; loui.husinat@yu.edu.jo (L.A.-H.); zaid.modanat@yu.edu.jo (Z.A.M.); 2 Faculty of Medicine, Yarmouk University, Irbid 21163, Jordan; bassiljoureyah978@gmail.com (B.J.); ahlamrawashdeh00@gmail.com (A.R.); abdalrahmanenizat@gmail.com (A.A.); 3 Anesthesia and Intensive Care Unit, Salmanyeh Hospital, Manama 323, Bahrain; abushihabmohammad@yahoo.com; 4 Department of General Surgery and Anesthesia, Faculty of Medicine, Mutah University, Karak 61710, Jordan; moh.amir@mutah.edu.jo; 5 Anesthesia and Intensive Care, IRCCS Ospedale Policlinico San Martino, 16132 Genova, Italy; 6 Department of Anesthesia and Intensive Care, University of Foggia, 71122 Foggia, Italy; gilda.cinnella@unifg.it

**Keywords:** high-flow nasal cannula, high-flow nasal oxygen, perioperative setting, non-invasive mechanical ventilation, acute hypoxemic respiratory failure, procedural sedation

## Abstract

High-flow oxygen therapy (HFOT) is a respiratory support system, through which high flows of humidified and heated gas are delivered to hypoxemic patients. Several mechanisms explain how HFOT improves arterial blood gases and enhances patients’ comfort. Some mechanisms are well understood, but others are still unclear and under investigation. HFOT is an interesting oxygen-delivery modality in perioperative medicine that has many clinical applications in the intensive care unit (ICU) and the operating room (OR). The purpose of this article was to review the literature for a comprehensive understanding of HFOT in the perioperative period, as well as its uses in procedural sedation. This review will focus on the HFOT definition, its physiological benefits, and their mechanisms, its clinical uses in anesthesia, and when it is contraindicated.

## 1. Introduction

Various types of respiratory support systems are applied to enhance oxygenation and ventilation in patients experiencing acute hypoxemic respiratory failure (AHRF) in the perioperative period. Among these, there are low-flow oxygen-delivery systems such as nasal cannulas or facial masks and high-flow-oxygen-delivery systems such as venturi masks or non-rebreathers. High-flow oxygen therapy (HFOT) is a respiratory support system that is gaining growing attention worldwide [1]. HFOT is when high flows of heated and humidified gas are administered to the upper airways at flow rates that are higher than those provided by conventional oxygenation methods. A high flow of heated and humidified air can be utilized either independently or in conjunction with oxygen in order to produce varying levels of inspiratory oxygen fractions (FiO_2_) that span from 0.21 to 1 [2]. HFOT demonstrated serious advantages in improving gas exchange and enhancing patients’ tolerance to oxygen therapy, sometimes reducing the rate of re-intubation [3]. 

## 2. Methods

In this literature review, we conducted a comprehensive search using PubMed and Google Scholar, from inception to September 2023, to find all relevant articles using different keywords such as “high flow oxygen therapy”, “tracheostomy”, “non-invasive ventilation”, “preoxygenation”,” perioperative”, “extubation”, and “sedation”. We applied filters to exclude studies that were conducted in languages other than English and studies in patients younger than 18 years.

## 3. From Conventional Oxygen Therapy to High-Flow Oxygen Therapy

Conventional oxygen therapy (COT), which provides oxygen through standard nasal cannulas and venturi/reservoir masks, has long been regarded as the primary treatment for AHRF. However, through COT, limited oxygen flow (up to 15 L/min) can be administered due to insufficient humidification and heating of the inhaled gas [4]. During inspiration in normal breathing, the air is heated up to body temperature and naturally humidified with water vapor as it moves from the nose down to the alveoli. Although medical gas provided to patients who are spontaneously breathing is occasionally humidified with bubble humidifiers, the absolute humidity of the emergent gas remains low [2,5]. Poorly humidified gas leads to poor tolerance of oxygen therapy because it may cause dryness of the nose and throat and nasal pain. Also, unconditioned gas increases airway resistance, and this is by reducing the flow in the upper airways to protect the lung from the dry cold inspired air [4,6]. Moreover, it is known that breathing dry air decreases nasal mucociliary clearance [7]. Additionally, the low flow of oxygen (15 L/min) delivered by COT is far less than the required inspiratory flow required by patients with AHRF, which ranges from 30 to >120 L/min [8]. 

Noninvasive mechanical ventilation (NIV) is a more advanced approach than COT for improving ventilation, enhancing gas exchange, and reducing the work of breathing [9]. NIV has become the chosen primary modality for respiratory support in patients with acute exacerbations of chronic obstructive pulmonary disease (COPD) because it increases inspiratory tidal volume (V_T_) and ensures sufficient alveolar ventilation is maintained [10]. However, although effective, NIV may be uncomfortable, and even frightening, for some patients, due to the high pressures delivered to the airways, difficulty synchronizing breathing, stomach distention, claustrophobia, and other mask-related side effects, such as nose sores and skin lesions over the bridge of the nose [11,12].

**Figure 1:**
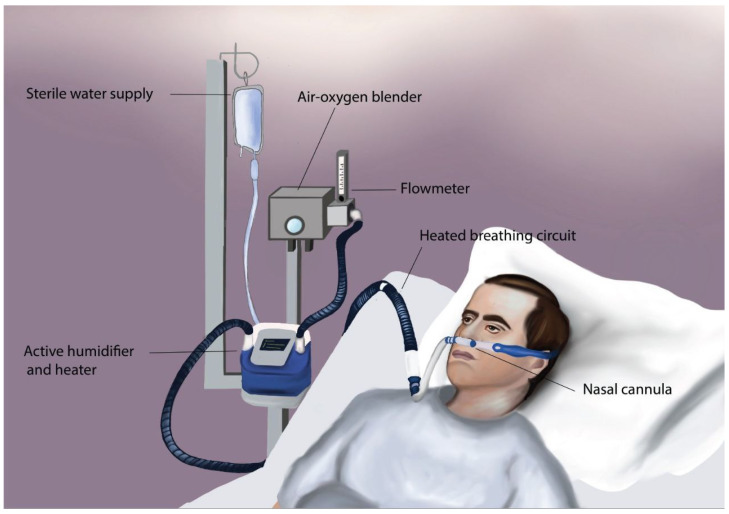
An illustration of one of the techniques by which HFOT can be administered, showing a nasal cannula device, which consists of an air–oxygen blender, allowing for FiO_2_ ranging from 0.21 to 1.0 and a flowmeter that generates up to 60 L/min flow. The gas is then humidified and heated in the active humidifier and heater and is delivered via the heated breathing circuit to the patient (i.e., through a soft wide-bore nasal cannula).

More recently, new devices and methods that provide heated and humidified high flows of oxygen emerged as alternatives to NIV in many clinical settings. HFOT can be administered via nasal cannulas or tracheal cannulas via dedicated devices such as the nasal cannula device or intensive care unit (ICU) ventilators [13,14]. See Figure 1.

## 4. Mechanisms of Action of HFOT

The advantages of employing HFOT are manifold. Firstly, when administered through nasal cannulas, this method provides a simple and comfortable means of delivering oxygen, eliminating the need for a face mask. This allows patients to eat, drink, and speak while receiving therapy, enhancing overall convenience. Additionally, HFOT confers various benefits to the respiratory system, some of which have been extensively studied, while others are not yet fully understood [14].

One of the primary benefits is the delivery of oxygen at high flow rates (up to 60 L/min), which effectively reduces the volume of anatomical dead space by flushing out exhaled carbon dioxide from the upper airways. This minimizes the re-breathing of carbon dioxide (CO_2_) [15,16]. Furthermore, the high flow rates prevent significant mixing with room air, ensuring a closer match between the supplied and designated fraction of inspired oxygen (FiO_2_) [17]. Moreover, it promotes an enhanced breathing pattern characterized by a lowered respiratory rate and an increased tidal volume [1,18,19]. This effect combined with the preconditioned oxygen, which is preheated to 37 °C and humidified to a level of 44 mg H_2_O/L (100% relative humidity) by the nasal cannula device, eases resistance during inhalation, resulting in decreased respiratory effort and lower metabolic demand on the patient. Consequently, the work of breathing (WOB) is minimized [20,21].

Additionally, while HFOT is not a closed system, the substantial flow rate it generates restricts air outflow during exhalation and elevates airway pressure [22]. This leads to a greater volume of air remaining in the lungs post exhalation compared to low-flow oxygen therapy. The application of positive pressure assists in the recruitment of alveoli and lessens the mismatch between ventilation and perfusion [21,23,24]. See Table 1.

The physiological benefits of HFOT may vary depending on whether it is delivered via a nasal cannula or a tracheal cannula. The results of a crossover study by Natalini et al. revealed that while HFOT via tracheal cannula moderately improves oxygenation and reduces respiratory rate, a minimum flow rate of 50 L/min is necessary to achieve these effects. In contrast, HFOT through a nasal cannula can accomplish similar benefits at a lower flow rate of 30 L/min. This suggests that in tracheal delivery, the reduced anatomical dead space and inspiratory resistance call for higher flow rates in order to exert a significant impact. Additionally, the enforced unidirectional flow of both inhalation and exhalation clears anatomical dead space and enhances respiratory efficiency. This action aids in CO_2_ elimination regardless of the specific oxygen therapy device employed, thereby lessening the influence of HFOT via tracheostomy. Furthermore, the study demonstrates higher expiratory pressure levels when high flows of oxygen were delivered using a nasal cannula compared to when delivered via tracheostomy. This indicates that the generation of expiratory pressure does not solely depend on the high oxygen flow rates but also on factors like the increased resistance posed by the upper airways. Hence, in patients with a tracheostomy, where resistance is minimal, the generated pressure is relatively lower [25].

## 5. Clinical Uses of HFOT in the Perioperative Period

### 5.1. The Role of HFOT in the Preoperative Setting

#### 5.1.1. Rationale

Surgical interventions often require general anesthesia with endotracheal intubation (ETI). ETI is a procedure that is performed to administer air, oxygen, and even inhalational anesthetics to patients during surgical operations and in the perioperative setting. ETI is not free of complications, and hypoxygenation with desaturation represents one of the most fearful complications of this procedure.

To avoid the complications associated with the ETI procedure, pre-oxygenation, which is the delivery of 100% FiO_2_ pre-intubation, has been a common practice to extend the non-hypoxic apnea time, the so-called margin of safety [26]. Prior to general anesthesia, it is generally recommended to oxygenate patients with healthy lungs through a facial mask [6]. However, patients who have increased metabolic demand for oxygen or reduced functional residual capacity will have a limited oxygen reservoir and a reduced time to desaturation. In these patients, extending the apnea time without desaturation before intubation is necessary for the management of a difficult airway. Bariatric, obstetric, septic, and hypoxemic patients due to any underlying cause represent potential groups where using NIV, alone or accompanied by HFOT, should be used instead of conventional facial masks [27]. NIV’s beneficial effects lie in the fact that it delivers positive pressure to the alveoli and reduces or reverses alveolar collapse and atelectasis formation, thus improving oxygenation and ventilation and preventing eventual hypoxemia [28]. Although NIV can be used to pre-oxygenate patients, it has to be stopped when a laryngoscopy is performed. As a consequence, during ETI, NIV cannot completely prevent desaturation. As an alternative, HFOT via nasal cannula can be applied to preoxygenate patients because it does not interfere with laryngoscopy [29]. In addition, HFOT has been successfully used in awake fiberoptic intubation, where a major advantage appears to be its ability to deliver an FiO_2_ close to 1.0 via soft nasal cannulas allowing the passage of a fiberoptic scope. It was safe, tolerated well, and enhanced oxygenation while steering clear of hypercapnia [30].

#### 5.1.2. Clinical Evidence in Hypoxemic Patients

There is conflicting evidence regarding pre-oxygenating hypoxemic patients before intubation through HFOT. Jaber et al. conducted a randomized controlled trial, in which they compared 4 min of pre-oxygenation through HFOT accompanied by NIV with pre-oxygenating patients with NIV alone in severely hypoxemic ICU patients with respiratory failure. The results showed that using HFOT or NIV for pre-oxygenation led to higher arterial partial pressure of oxygen (PaO_2_) and a lower percentage of patients experiencing drops in oxygen levels below 80% (0% vs. 21%). However, the number of patients involved was low, and small-sized studies tend to overestimate the treatment effect [31]. Conversely, in a randomized controlled trial conducted by Vourc’h et al., researchers evaluated the efficiency of using HFOT versus conventional high-flow oxygen face masks in pre-oxygenating 119 severely hypoxemic adults, they concluded that HFOT did not reduce the lowest peripheral oxygen saturation (SpO_2_) when compared to using conventional masks [32]. Guitton et al. reported similar results when they evaluated pre-oxygenating 184 non-severely hypoxemic patients in the ICU with HFOT compared to bag-valve-mask oxygenation during rapid sequence intubation (RSI). They found that pre-oxygenating ICU patients with HFOT led to a decreased incidence of intubation-related adverse events but did not improve the lowest SpO_2_ compared with standard mask oxygenation. The varying outcomes could be attributed to dissimilarities among the studies in terms of intubation indications and the level of hypoxemia severity before intubation [33].

In light of the available evidence, it is evident that HFOT did not provide significant advantages on SpO_2_ in severely and non-severely hypoxemic patients in comparison with conventional mask oxygenation. However, it decreased the incidence of complications related to intubation by threefold, which is a significant finding given the high complication rate (over 10%) even in non-severely hypoxemic patients [33].

#### 5.1.3. Clinical Evidence in Obese Patients

Obese patients carry a higher risk of difficult mask ventilation and difficult tracheal intubation when compared with non-obese patients [34]. Moreover, in obese patients, lung volumes and lung compliance are decreased; consequently, the functional residual capacity (FRC) is also decreased [35]. In the perioperative period, these situations are exacerbated, and this results in a higher risk of hypoxemia in these patients. As a consequence, appropriate oxygen therapy is critical to the prevention of perioperative complications in these patients [36].

In a randomized trial published in 2022, Wu et al. compared the oxygenation efficacy between 80 obese patients who were oxygenated through HFOT (*n* = 40) versus standard facemasks (*n* = 40) during elective tracheal intubation. They found that HFOT oxygenation achieved a higher PaO_2_ compared to facial mask oxygenation and that the desaturation risk of peri-intubation was significantly less in the HFOT group compared to the face mask group [37]. In a recent randomized controlled trial published in 2023, Schutzer-Weissmann et al. compared the duration of apnea between obese patients who received high-flow nasal oxygen (*n* = 41) and others who received low-flow facemask oxygen (*n* = 39). In their study, it was observed that most patients did not experience oxygen desaturation for 18 min, regardless of whether oxygen was delivered through HFOT at 70 L/min or a low-flow facemask at 15 L/min. However, the use of high-flow nasal oxygen appeared to lower the desaturation risk when compared to facemask oxygen delivery. Notably, the risk of desaturation is a more clinically significant outcome than the duration of apnea. Furthermore, their conclusion emphasized that the primary determinant of desaturation risk is likely to be individual patients’ physiological factors rather than the specific method of oxygen delivery [38]. In another randomized controlled trial published in 2021, Rosén et al. compared the efficacy of using HFOT in comparison to facial masks with positive end-expiratory pressure (PEEP) for pre-oxygenating 40 obese patients, and they concluded that HFOT provided adequate pre-oxygenation quality in all subjects and may be considered as an alternative to a face mask in selected patients. However, using a facemask with peep was superior to HFOT for pre-oxygenating obese patients [39]. Having found conflicting evidence, in 2023, Zhou et al. conducted a systematic review and meta-analysis of 12 trials comparing HFOT with COT, with a total sample of 798 obese patients in the perioperative period. Their results revealed a 60% reduction in the risk of hypoxemia when using HFOT compared to COT, improved SpO_2_ levels, lessened need for additional respiratory support, and therefore reduced patients’ length of stay (LOS) in the hospital [36].

The systematic review by Zhou et al. confirmed that the current literature favors the use of HFOT in the perioperative period in obese patients. However, further large-scale trials are needed to confirm HFOT’s efficacy in obese patients. Consistency in intervention timing, obesity levels, surgery types, hypoxemia definitions, and respiratory support thresholds should be investigated in future clinical trials [36].

#### 5.1.4. Clinical Evidence in Pregnant Patients

During pregnancy, the mother’s body demands more oxygen and FRC is lower than usual, due to the upward displacement of the diaphragm caused by the gravid uterus. Whereas it generally takes 243 s for the SpO_2_ to drop to 95%, the time for a term pregnant woman is only 173 s. This means that in case the mother needs general anesthesia during delivery, she must receive sufficient pre-oxygenation to minimize the risk of hypoxemia [40].

According to current guidelines, it is recommended that anesthesiologists use HFOT or standard nasal cannula for apneic oxygenation of pregnant women during airway instrumentation [41]. However, these guidelines were based on findings that were inferred from studies on non-pregnant patients. When the guidelines were formed, evidence on the usage of high-flow nasal oxygen for pre-oxygenating pregnant women was rare. However, some studies with contradicting results were conducted after that [42]. In a study conducted by Tan et al. investigating the use of HFOT in term pregnant women, pre-oxygenation with HFOT was not adequate as an end-tidal EtO_2_ of ≥90% could not be reached after 3 min. They think that achieving adequate outcomes would take more time [43]. In a 2020 study conducted by Au K et al., the researchers, through a biased coin up-down allocation trial, reached the conclusion that even after 8 min of pre-oxygenation with an HFOT, it is not possible to attain EtO_2_ levels of 90% or higher. They additionally validated the findings of Tan et al., reaffirming that using a face mask for 3 min is insufficient for adequately pre-oxygenating pregnant patients. The primary factor leading to inadequacy is the trapping of air. Obtaining an EtO_2_ ≥ 90% might not be feasible for term pregnant women undergoing cesarean delivery [44].

While previous studies have labeled HFOT as an unsatisfactory pre-oxygenation technique due to its inability to achieve the desired oxygenation target of 90% or more, more recent research emphasizes the importance of focusing on the time to desaturation as a preferred objective. Using computational modeling (Nottingham physiology simulator) conducted by Pillai et al. showed that HFOT substantially extends safe apnea time in pregnant patients [45]. Additionally, Zhou et al. in a randomized, unblinded trial, compared the use of a standard face mask with HFOT in 34 healthy mothers being prepared to undergo a cesarean section under general anesthesia for pre-oxygenation and apneic oxygenation. Although the study revealed that the two groups were similar with regard to PaCO_2_ values, the lowest level of saturation, duration of apnea, pH value, intubation times, and fetal outcomes regardless of the oxygenation method used, the authors concluded that HFOT was the better option, as it resulted in higher PaO_2_ and EtO_2_ levels immediately following intubation [40]. Hence, when factoring in both pre-oxygenation and apneic oxygenation, the duration of safe apnea achieved by oxygenating patients with HFOT may surpass that of the conventional face mask method [40,42]. Moreover, a recent study examined the duration of safe apnea in parturients undergoing cesarean sections under general anesthesia. The study randomly assigned 100 patients to receive pre-oxygenation with HFOT via nasal cannula or with the traditional facemask and a nasal cannula delivering a low flow of oxygen during apnea. Following pre-oxygenation, all patients were induced with general anesthesia. Although immediate intubation was performed in all cases, ventilation commenced only when oxygen saturation fell below 90%. The study revealed a significant difference in the median safe apnea duration between the HFOT group (7 min) and the facemask group (4 min) [46]. In a more recent non-randomized pilot study, Sjöblom et al. focused on parturients undergoing cesarean sections under general anesthesia, comparing the effects of pre and peri-oxygenation with HFOT via nasal cannula to traditional facemask pre-oxygenation. The primary outcome measured was the proportion of parturients experiencing desaturation below 93% during the procedure. No cases of desaturation were observed in either group (total of 34 parturients), with the lowest peripheral oxygen saturation recorded at 97%. There were no significant differences in end-tidal oxygen concentrations, suggesting that HFOT effectively maintains oxygen saturation levels during anesthesia induction in parturients [47].

Building on the previous discussion, it appears that HFOT is not an inferior alternative to conventional oxygenation methods in pregnant patients. However, to determine whether it offers superior effects, further large-scale randomized clinical trials should be conducted, taking into account the limitations of the few clinical trials on this subject.

### 5.2. The Role of HFOT in the Postoperative Setting

#### 5.2.1. Rationale

Roughly 234 million surgical procedures are performed annually, with approximately 1.3 millions of these patients experiencing post-surgery complications [48]. In the period immediately following surgery, the patient is at risk of pulmonary complications, hypoxemia being the most prevalent (between 30 and 50%) [49]. Acute respiratory failure can occur if oxygen levels fall after tracheal extubation. This can lead to the patient needing re-intubation [50]. Thus, all patients are oxygenated after operation, which is usually conducted using low-flow systems. When high-flow systems are required, venture masks or high-flow nasal cannulas are typically used. However, when the patient is intubated, a tracheal cannula is used instead. The selection of a respiratory support system should be tailored to each individual’s needs and relies on various factors, such as the level of oxygen needed, the cause of respiratory failure, and the preferences of the patient [1,13].

After extubation, providing oxygen is the most used treatment to correct hypoxemia. Post-operatively, using NIV may pose challenges due to inadequate facilities or resources in certain settings (such as general surgical wards) or the patient’s inability to tolerate the interface. In contrast, HFOT via nasal cannulas offers fewer burdens for the patient and the caregiver and provides a less traumatic interface for the patient. However, a key drawback of HFOT is that it mainly treats hypoxemia by administering high flows of oxygen levels without treating atelectasis because it does not provide as high pressures as NIV does [2,51].

#### 5.2.2. Clinical Evidence Post-Cardiac Surgery

Due to the usage of cardiopulmonary bypass and perioperative blood transfusion, patients who undergo cardiovascular surgery should be oxygenated after extubation to maintain safe levels of oxygen in the blood and reduce the incidence of re-intubation. Stephan et al. conducted a randomized multicenter study on 35 patients, through which they assessed whether HFOT was less effective than NIV, specifically administered as bilevel positive airway pressure (BiPAP) in treating or preventing acute respiratory failure (ARF) following cardiothoracic surgery. They found out that HFOT administered via nasal cannulas is not an inferior option to NIV since the treatment failure rate was 21% in the HFOT group compared to 21.9% in the NIV. In addition, there was not a statistically significant difference in ICU mortality rate between the two groups. Moreover, nasal skin lesions were significantly observed more in the NIV group after 24 h [52]. A more recent randomized crossover trial conducted by Shiho et al. examined the short-term efficacy of HFOT vs. Venturi Mask (VM) when applied to a sample of 35 cardiovascular surgery patients. A total of 17 patients received oxygen via HFOT followed by VM (protocol A), while the remaining 18 patients were given VM followed by HFOT (protocol B). After 60 min of treatment, levels of PaO_2_ and PaCO_2_ in the protocol A group were deemed more favorable than those in protocol B. Also, there was no difference in the hemodynamic state between the two groups. Thus, the authors concluded that HFOT was suitable for use in cardiovascular surgical patients [49]. In a meta-analysis, Zhu et al. assessed the safety and efficacy of HFOT delivered through nasal cannulas compared to COT in 495 cardiac surgical patients post extubation. They found out that HFOT is associated with a significant decrease in escalating respiratory support. However, there were no statistically significant differences between the two treatment groups in reintubate rate or the length of ICU stay [53].

A review of the evidence reveals that HFOT administered via nasal cannulas can be safely used as a first option in cardiovascular patients after extubation. When compared to NIV and COT, it does not impede the prognosis in this patient population and has some advantages, such as reduced nursing workload and easy application. Moreover, it improves oxygenation and decreases the effort required by the patient during breathing compared to VM.

#### 5.2.3. Clinical Evidence Post-Thoracic Surgery

Postoperative pulmonary complications (PPCs) such as pneumonia, atelectasis, and hypercapnia are common after thorax surgery, especially lobectomy due to impairment of postoperative respiratory function. Additionally, hypoxemia following thoracic surgery is considered a life-threatening condition and is a leading cause of death in this patient population and that is due to many reasons such as the increased incidence of surgical pneumothorax, anesthesia-induced pulmonary atelectasis, and a decreased FRC caused by lung resection [54,55]. Therefore, respiratory support and oxygenation post-extubation are of great importance to prevent these complications and subsequent respiratory failure. While using conventional oxygenation methods such as nasal prongs or a facemask to provide oxygen can help supplement oxygen intake, they may not effectively compensate for lung volume loss or support proper gas exchange, especially in patients who have undergone lobectomy. Therefore, Yu et al. investigated if HFOT is superior to COT post-extubation for reducing hypoxemia and PPCs in patients who undergo thoracoscopic lobectomy. They concluded that HFOT enhances patients’ oxygenation and decreases the likelihood of re-intubation. However, it does not have any effects on PPCs [56]. Furthermore, in a more recent randomized trial, Pennisi et al. studied if HFOT decreases the incidence of postoperative hypoxemia in 95 patients who underwent thoracotomy lung lobectomy compared to VM oxygen therapy. They found out that HFOT provided by nasal cannulas did not reduce the incidence of postoperative hypoxemia or any other postoperative pulmonary complication [57].

Results emerging from trials comparing HFOT and COT performance postoperatively indicate that HFOT outperforms COT in terms of oxygenation for patients following thoracic surgery; however, HFOT may not have an effect on postoperative complications in this patient population. Moreover, the clinical effects of HFOT may be constrained when compared to VM. Further studies are warranted to confirm HFOT’s utility in this setting.

#### 5.2.4. Clinical Evidence Post-Abdominal Surgery

Patients who undergo major abdominal surgery have a high incidence of PPCs. In fact, the incidence of PPCs can be as high as 40% in patients who are American Society of Anesthesiologists (ASA) IV or patients who underwent surgeries to the thorax or the upper abdomen [58]. In addition, prophylactic postoperative NIV is not recommended, as it does not decrease the incidence of PPCs and makes patients more prone to gastrointestinal fistulas [59]. Therefore, in recent years, there has been a growing interest in studying the effect of HFOT in this patient population. In a multicenter randomized controlled trial, Futier et al. evaluated the use of HFOT in the prevention of hypoxemia in 220 patients who underwent major surgery to the abdomen. They concluded that the prophylactic use of HFOT post-extubation did not improve pulmonary outcomes in comparison with COT [60].

More recently, Jin et al. initiated a randomized controlled trial to evaluate whether HFOT combined with respiratory training would decrease PPC incidence compared to COT in patients undergoing major surgery to the abdomen. Currently, the study is underway, and the results are anticipated to provide the literature with some insights into the potential benefit of this approach for these patients. Further studies are required before reaching any definitive conclusions [61].

Liver transplantation surgeries represent a category of abdominal surgeries. Patients who undergo liver transplantation must endure a taxing surgical procedure because of its duration and the associated risk of bleeding. Because of the risk of bleeding, surgery may require massive transfusion, which can be associated with an increased risk of respiratory failure. Thus, oxygen delivery is recommended postoperatively to prevent its complications. Gaspari et al. conducted a matched controlled study in which they compared the preventive use of HFOT versus standard O_2_ (air-entrainment mask) post-extubation in adults who underwent liver transplantation. They found that early application of HFOT in patients with liver transplantation did not decrease the incidence of hypoxemia post-extubation compared with standard O_2_ and did not have an effect on the incidence of weaning failure, ICU length of stay, and 28 days mortality in this high-risk population of patients [62].

To our knowledge, there are no more studies assessing the effect of HFOT in preventing hypoxemia post-extubation in individuals who underwent liver transplantation surgery. Further studies are needed to draw a conclusion on the safety and efficacy of HFOT post-transplant surgery.

Lastly, having found conflicting evidence in the literature, Zhu et al. conducted a systematic review and meta-analysis of 10 studies that compared the use of COT with HFOT post-extubation; 856 patients were in the HFOT group, while the COT group comprised 852 patients. These patients included post-cardiac surgery, major abdominal surgery, ICU, or AHRF patients who were scheduled for planned extubation. The findings showed that HFOT, in comparison to COT, may significantly reduce post-extubation respiratory failure and respiratory rates and increase PaO_2_ [63]. A similar review and analysis carried out by Granton et al. made similar findings, concluding that, although HFOT after extubation resulted in a decrease in re-intubation rates and compared with COT, it had no advantage over NIV except for shortened ICU admission and increased patient comfort [64].

## 6. Clinical Uses of HFOT in Procedural Sedation

Sedation can be described as a decrease in consciousness level brought about by medication, often used in procedures that may cause the patient apprehension or pain. It can also serve to reduce the patient’s recollection of the experience [65]. Furthermore, the use of sedation and pain relief can aid in significantly enhancing the swift and effective execution of medical procedures. However, it is important to note that any of the medications used for sedation and analgesia can cause negative side effects. These undesirable effects typically impact respiratory function or the cardiovascular system [66].

### 6.1. The Role of HFOT during Sedated GI Endoscopy

In general, gastrointestinal endoscopic (GIE) procedures are considered safe and are widely utilized due to their minimally invasive approach, which allows for both the accurate diagnosis and treatment of various conditions [67]. Today, approximately 50% of endoscopic procedures are performed under monitored anesthesia care (MAC) to deeply sedate the patient [65]. However, as previously stated, there are risks associated with sedation. Among the potential pulmonary complications are respiratory depression caused by commonly used sedative drugs, as well as airway obstruction resulting from anatomical structures or foreign bodies. These conditions can lead to hypoxemia, which is the most prevalent cardiorespiratory complication during endoscopy. Hypoxemia can give rise to various adverse effects. Consequently, the utilization of supplemental oxygen is prevalent during GIE procedures [67].

Hypoxemia is a possible complication of gastroscopy procedures. Severe hypoxia can result in interrupting the procedure, needing mask ventilation, or even endotracheal intubation. Prolonged hypoxemia can lead to a range of detrimental complications, including cardiac arrhythmias, ischemia, and in some cases death [68,69]. A study by Bell et al. demonstrated that oxygen supplementation through nasal cannula during gastroscopy procedures was effective in reducing the incidence of hypoxemia from 77% to 16%. For this reason, the HFOT approach is now widely adopted for gastroscopy procedures [70].

Lin et al. conducted a multicenter RCT, to evaluate the efficacy and safety of supportive HFOT in preventing hypoxia during sedated gastroscopy. The study involved 1994 patients divided into two groups: Group 1 received supportive oxygen via nasal cannula at a flow rate of 2 L/min, while Group 2 received supportive HFOT via nasal cannulas at flow rates ranging from 30 to 60 L/min. Authors concluded that HFOT not only reduces hypoxemia events as compared to COT, but also reduced severe hypoxemia incidence in patients classified as Class I and II according to the American Society of Anesthesiologists (ASA), and underwent elective gastroscopy with propofol sedation. Furthermore, HFOT demonstrated minimal adverse effects and excellent patient tolerance [71].

In support of these findings, another RCT by Nay et al. was conducted to investigate whether the use of HFOT via nasal cannula could diminish the occurrence of reduced oxygen saturation levels (SpO_2_ ≤ 92%) when compared to standard oxygenation methods in patients undergoing gastrointestinal endoscopy with deep sedation. All patients included were adults with a moderate-to-high risk of experiencing hypoxemia. Among the patients subjected to HFOT, only 9.4% (18 out of 191) exhibited SpO_2_ levels falling below 92% during the procedure. In contrast, the proportion was notably higher at 33.5% (63 out of 188) among those receiving oxygen via standard methods of administration. Additionally, the occurrence of prolonged desaturation (lasting more than one minute) as well as the need for interventions to maintain open upper airways was less frequent in the HFOT group [72].

Endoscopic retrograde cholangiopancreatography (ERCP) is a frequent gastrointestinal endoscopy procedure that plays a vital role in the management of biliary and pancreatic conditions [73]. Unfortunately, ERCP is associated with a heightened risk for hypoxemia and hypoventilation due to several contributing factors. Firstly, the patient population requiring ERCP is often of advanced age. Furthermore, ERCP necessitates deeper levels of sedation compared to other GI endoscopy procedures, partly owing to its more invasive nature [74,75]. Additionally, patients are often placed in a prone position during the intervention, which reduces the degree of difficulty of the procedure but can further accentuate the challenges associated with maintaining optimal oxygenation levels [76]. These factors underscore the importance of careful airway management and monitoring during ERCP procedures.

An RCT by Kim et al. investigated the effectiveness of administering HFOT via nasal cannula in maintaining patients’ oxygenation levels during ERCP in the prone position, as compared to conventional oxygen therapy. HFOT was superior to conventional oxygen administration in maintaining acceptable oxygenation levels during ERCP procedures in the prone position. Furthermore, in the HFOT group, there was no incidence of hypoxemia or hypercapnia episodes, and the lowest recorded SpO_2_ was 98%, eliminating the need for airway interventions during the procedure [77].

To further strengthen the evidence and offer a comprehensive perspective, a very recent systematic review and meta-analysis by Khanna et al. (2023) examined the benefits of HFOT as opposed to COT during upper gastrointestinal endoscopies with sedation. The study included data from eight controlled studies and one longitudinal study, encompassing a total of 3294 patients. The aim was to assess the impact of HFOT on the duration of the procedures, interruption rates, occurrences of hypoxia, lowest SpO_2_ levels, complications, total propofol dosage, and the satisfaction levels of both patients and endoscopists. While the findings suggested that the HFOT group experienced improved oxygenation, fewer procedural interruptions, and higher nadir SpO_2_ levels, it is worth noting that the quality of the evidence was questioned because there was great variation between the studies regarding population and setting [78].

In conclusion, existing studies clearly indicate that HFOT has its advantages, such as potential benefits in mitigating hypoxemia and enhancing compliance among both endoscopists and patients that can be attributed to the preconditioning of the oxygen delivered [77]. However, due to the conflicting evidence regarding the effectiveness, more standardized studies focused on larger samples are warranted to provide clearer insights into the role of HFOT and its optimal use in sedated GI procedures.

### 6.2. The Role of HFOT during Sedated Bronchoscopy

Fiber optic bronchoscopy (FOB) and endobronchial ultrasound (EBUS) are both indispensable techniques in diagnosing a variety of pulmonary conditions such as lung infections, tumors, inflammation, and other lung and mediastinal diseases [79,80]. During bronchoscopy with sedation, patients can encounter challenges such as hypoxemia and respiratory depression, stemming from the combined effects of sedation and bronchial occlusion. These factors result in reduced respiratory drive, leading to hypoventilation. To mitigate these risks, it is crucial to provide oxygen supplementation during the bronchoscopy procedure [81].

Patients diagnosed with pulmonary lesions often experience hypoxemia, possibly complicating the procedure of flexible bronchoscopy (FB). Saksitthichok et al. conducted a randomized prospective study to compare the use of HFOT via nasal cannula to NIV in 51 patients needing FB. The aim was to discover which method was most effective in maintaining oxygen saturation during the procedure. HFOT and NIV yielded similar results in preventing hypoxemia. Further subgroup analysis indicated that in patients with initial PaO_2_ < 60 mmHg while breathing ambient air, NIV maintained oxygen levels more consistently compared to HFOT [82].

Another RCT, conducted by Ben-Menachem et al. (2020), examined the outcomes of using high-flow nasal oxygen or low-flow nasal oxygen for patients who had recently undergone lung transplants and were scheduled to have a transbronchial lung biopsy (TBLB) requiring FB. HFOT was found to reduce the number of patients who suffered from both mild and severe desaturation during FB, as well as those needing procedure interruptions and airway interventions [83].

The past 20 years have seen the increasing use of endobronchial ultrasound-guided transbronchial needle aspiration (EBUS-TBNA) as a way of evaluating various cancer-related conditions, diseases such as sarcoidosis and tuberculosis, and blood abnormalities such as lymphoma [84]. Irfan et al. conducted an RCT on 40 patients who underwent EBUS to determine their stage of lung cancer or diagnose abnormal findings in the mediastinum. Half of the sample received HFOT via nasal cannulas and the remaining half via conventional nasal prongs. The use of HFOT afforded significantly higher levels of oxygenation, a result that promises an enhanced safety profile for physically weak patients undergoing EBUS under moderate conscious sedation [85].

A systematic review and meta-analysis conducted by Sampsonas et al. encompassed six randomized controlled trials, with four of these trials employing intravenous sedation, while the other two utilized topical sedation. The objective of the study was to investigate the benefits of HFOT when applied during bronchoscopy procedures. The study highlighted the possibility that HFOT via nasal cannula is superior to low-flow nasal cannula in decreasing the incidence of hypoxemia and procedure interruption [80]. A similar systematic review conducted by Roy et al. (2022) of nine randomized controlled trials compared HFOT to various other modes of oxygen delivery, including low-flow devices, venturi masks, and NIV. The evidence was of low quality, primarily due to a noticeable lack of direct alignment between the characteristics of the study populations and the specific measurements or outcomes assessed in each study. Nevertheless, patients in the HFOT group achieved better oxygenation and experienced fewer desaturation events when compared to those using low-flow devices. However, when compared to NIV, the outcomes remained unclear [86].

The findings from the two reviews provide evidence that HFOT may offer significant benefits during bronchoscopy procedures with sedation. The results suggest that HFOT, compared to low-flow devices, can effectively reduce the occurrence of hypoxemia and interruptions during the procedure, as well as achieve better oxygenation levels. However, it is important to note that the quality of evidence varied and was sometimes limited. Overall, these findings indicate that HFOT has the potential to enhance outcomes when sedation is required during bronchoscopy. More inclusive research is necessary to determine its effectiveness in patients with comorbidities or high pulmonary risk.

### 6.3. The Role of HFOT during Sedated TAVR

Aortic valve stenosis (AS) is the most commonly encountered valvular disease [87]. The mainstay treatment for AS is aortic valve replacement, which typically involves open-heart surgery [88]. However, an increasingly preferred and less invasive approach, known as transcatheter aortic valve replacement (TAVR), is now favored for treating AS, especially among older patients and those with significant comorbidities. The transfemoral approach is the favored and most commonly chosen access site [89]. Transfemoral TAVR does not require sternotomy and is often performed with conscious sedation instead of general anesthesia [90], which has been observed to enhance hemodynamic stability and reduce hospital and ICU length of stay [91]. Nonetheless, due to the advanced age and presence of multiple comorbidities in most of the patients undergoing this procedure, there is a potential risk of hypoxemia. Furthermore, the requirement for patients to lie supine for approximately two hours during the procedure, along with sedation reducing their FRC, can contribute to potential complications [92].

A randomized controlled trial was conducted by Scheuermann-Jahn et al. to assess the efficacy of HFOT in enhancing oxygenation during sedated TAVR procedures compared to standard oxygen-delivery methods. One way that HFOT is known to improve oxygenation is through positive airway pressure, which is more pronounced when patients keep their mouths closed. However, contrary to their expectations, the researchers did not detect significant improvements in arterial oxygenation with HFOT, which the authors speculated was possibly due to patients keeping their mouths open during the procedure or other factors affecting sedated patients. They suggest that the enhanced oxygenation seen in other medical procedures like bronchoscopy, gastroscopy, and endoscopic retrograde cholangiopancreatography may be attributed to the placement of an endoscope, mimicking the effect of a closed mouth. Nonetheless, the HFOT group experienced a lower incidence of desaturation and airway manipulation, and they reported higher scores for comfort during the procedure [92].

In summary, it is essential to acknowledge the limitations of this trial, such as the relatively small sample size and suboptimal design for accurately evaluating the impacts of HFOT on secondary outcomes during TAVR procedures with sedation. In addition to the scarcity of existing literature on HFOT in this specific setting, these observations emphasize the need for larger, more in-depth, and precisely designed research to thoroughly assess the potential benefits, if any, of HFOT in enhancing the management of sedated patients undergoing TAVR. Expanding our understanding in this area can provide valuable insights to optimize the care of sedated patients during TAVR procedures.

## 7. Contraindications

Contraindications related to HFOT use in adults are uncommon. In general, there are no absolute contraindications because, to our knowledge, there are no randomized controlled trials that have set contraindications as the primary endpoint. However, relative contraindications are conditions that may hinder the proper fitting of a nasal cannula including surgical interventions, nasal or facial abnormalities, or experiencing claustrophobia. Additionally, it should not be used in patients who have respiratory arrest, are hemodynamically unstable, have a significant amount of secretions in their airways, are at increased risk of aspiration, are uncooperative, or with consciousness impairment [5]. Moreover, some experts refrain from using HFOT in individuals who have undergone surgery to the upper airway to prevent the potential theoretical possibility of elevated pressure leading to venous thromboembolism [1].

## 8. Conclusions

HFOT is a high-flow respiratory support system in which heated and humidified oxygen is delivered to the patient at a set flow rate via different interfaces. This system is growing in popularity and is increasingly used in a wide variety of applications in the ICU and operatory room. There are many beneficial effects of HFOT over COT devices or NIV, such as the reliable delivery of FiO_2_, the PEEP effect, the washout of nasopharyngeal dead space, improved comfort, and enhanced expectoration due to greater humidification of secretions; thus, it can replace COT in acute respiratory failure to avoid escalation to more invasive oxygen-delivery systems. Moreover, HFOT at least seems to be a non-inferior choice when compared to NIV and COT in some clinical settings in the perioperative period. In addition, it has some advantages during sedated bronchoscopy and endoscopy. However, its indications are not absolute, and further research is needed to gain robust evidence.

## Figures and Tables

**Table 1:** Summary of the physiological benefits of HFOT and their mechanisms.

Mechanism	Benefits
Anatomical dead space washout	▪ Enhances the effectiveness of ventilation. ▪ Minimizes re-breathing of CO_2_.
High oxygen flow rates	▪ Minimal admixture with room air. ▪ Delivery of a constant FiO_2_. ▪ Decreased work of breathing.
PEEP effect	▪ Recruitment of alveoli. ▪ Decreases ventilation perfusion mismatch.
Delivery of heated and humidified gas	▪ Minimizes airway constriction. ▪ Reduces effort required for breathing.
Administering oxygen through a silicone cannula	▪ Allows patients to eat, drink, and speak. ▪ Improved patient comfort.

PEEP = positive end-expiratory pressure; CO_2_ = carbon dioxide; FiO_2_ = fraction of inspired oxygen.

## Data Availability

Not applicable.
